# Effect of *Vetiveria zizanioides* Essential Oil on Melanogenesis in Melanoma Cells: Downregulation of Tyrosinase Expression and Suppression of Oxidative Stress

**DOI:** 10.1155/2014/213013

**Published:** 2014-03-19

**Authors:** Hsin-Yi Peng, Chin-Chun Lai, Chih-Chien Lin, Su-Tze Chou

**Affiliations:** ^1^Department of Food and Nutrition, Providence University, No. 200, Section 7, Taiwan Boulevard, Shalu, Taichung 43301, Taiwan; ^2^Department of Cosmetic Science, Providence University, No. 200, Section 7, Taiwan Boulevard, Shalu, Taichung 43301, Taiwan

## Abstract

The major objective of this study was to estimate the hypopigmentation function of the essential oil from *Vetiveria zizanioides* (VZ-EO). Our results indicated that VZ-EO exhibits potent lipid peroxidation inhibitory activity to moderate the bleaching of **β**-carotene and to maintain the cellular glutathione (GSH) levels. VZ-EO can markedly decrease melanin production and tyrosinase activity in **α**-melanin-stimulating-hormone- (**α**-MSH-) stimulated B16 cells. The effect of VZ-EO on melanogenesis is achieved by the suppression of cellular tyrosinase expression. The results demonstrated that the activity of VZ-EO on melanogenesis might be the result of its potent antioxidative ability, which was reflected in the decreased cellular oxidant and malondialdehyde (MDA) levels and the recovered activities of superoxide dismutase (SOD), glutathione peroxidase (GPX), and catalase (CAT) in **α**-MSH-stimulated B16 cells. The most abundant compound in VZ-EO is cedr-8-en-13-ol (12.4%), which has a strong capability to inhibit lipid peroxidation. Therefore, VZ-EO has the potential to become an ingredient in future hypopigmentation drugs, foods, and cosmetics.

## 1. Introduction

Melanin is a pigment that is widely distributed in the skin surface, hair, retina, and adrenal medullae and is biosynthesized from tyrosine through the enzymatic oxidation of tyrosinase [1]. Melanogenesis is primarily stimulated by the ultraviolet- (UV-) induced *α*-melanin-stimulating hormone (*α*-MSH) in melanocytes [2]. Moreover, melanin is thought to play an important role in skin cancer prevention through the protection of cells from UV radiation. However, increased amounts of melanin are observed in a large number of skin diseases and affect skin esthetics [3]. Accordingly, skin-whitening agents from synthetic or natural resources are under development for both beauty and therapeutic purposes.

Tyrosinase (EC 1.14.18.1) is the key enzyme that acts in the initial step of melanin biosynthesis. This enzyme catalyzes the oxidation of L-tyrosine to 3,4-dihydroxyphenylalanine (DOPA) and of DOPA to dopaquinone, which is then processed to dopachrome and eumelanin in the absence of cysteine (Cys) [4]. In contrast, in the presence of cysteine, dopaquinone rapidly reacts with cysteine to yield cysteinyldopas, which are then oxidized to produced benzothiazine intermediates and ultimately pheomelanin [2]. Glutathione (GSH) and its precursor, cysteine, are well known to react with oxidative species in cells and play an important role in the cellular protection system against reactive oxidative species (ROS) [5, 6]. Therefore, the melanogenesis-mediated decrease of glutathione and cysteine is recognized as an oxidative stress for melanocytes [5].

*Vetiveria zizanioides* (vetiver grass), which is a perennial tussock grass of the Gramineae family, is famous as an ecofriendly plant. This grass can prevent soil erosion and is used in the treatment of metalliferous-polluted ground due to its tolerance to heavy metals [7].* V. zizanioides* has also been cultivated for many industrial applications, including the production of the commercially and medicinally valued volatile oil that can be distilled from its root [7, 8].* V. zizanioides* essential oil (VZ-EO) has been frequently used as a functional ingredient and fragrance in foods, aromatic products, and cosmetics. The vetiver oil is an expensive edible oil in the Chinese market and which has also been used in India in many ways as a food additive, such as flavoring syrups, ice cream, and beverages and for food preservation. Moreover, VZ-EO is commonly used as traditional medicine in Thailand and India for the treatment of numerous syndromes, such as gastritis, fever, headache, mouth ulcers, toothache, and chronic inflammation [9, 10].

In a previous study, we demonstrated that the anti-inflammatory activity of VZ-EO correlates with its antioxidant activity, which decreases the lipopolysaccharide- (LPS-) stimulated superoxide anion production and the malondialdehyde (MDA) levels in macrophages [11]. However, the antimelanogenic activity of VZ-EO has not been studied to date. Therefore, the major objective of this study was to estimate the hypopigmentation function of VZ-EO. The relationship between the antioxidant function and the antimelanogenic activity of VZ-EO in *α*-MSH-treated melanocytes was also investigated in this study.

## 2. Experimental

### 2.1. Chemical and Reagents

The steam distilled essential oil of* Vetiveria zizanioides* (VZ-EO) was purchased from Lorien Vana Biotech, Inc., Taiwan. The *α*-melanocyte stimulating hormone (*α*-MSH), dimethyl sulfoxide (DMSO), monobromobimane (MbBr), and phenylmethylsulfonyl fluoride (PMSF) were purchased from Sigma-Aldrich Chemicals Co. (St. Louis, MO, USA). The L-3,4-dihydroxyphenylalanine (L-DOPA) that was used in this study was purchased from Merck (Darmstadt, Germany). Dulbecco's modified eagle medium (DMEM), fetal bovine serum (FBS), L-glutamine, and penicillin-streptomycin were purchased from Invitrogen Life Technologies (Carlsbad, CA, USA). The goat antityrosinase antibody and rabbit anti-*β*-actin antibody were purchased from Santa Cruz Biotechnology (CA, USA). The enhanced chemiluminescence (ECL) kit was purchased from Amersham Biosciences (NJ, USA). All of the other chemicals that were used were at least of reagent grade. The deionized distilled water (ddH_2_O) used for the preparation of solutions and buffers was purified using a Milli-Q system (Millipore, Bedford, MA, USA).

### 2.2. Determination of Antioxidant Activity through the *β*-Carotene Bleaching Assay

The antioxidant activity was evaluated according to the *β*-carotene bleaching method [12]. First, 0.1 mg of *β*-carotene was dissolved in 5 mL of chloroform. One milliliter of the chloroform solution was mixed with 20 *μ*L of linoleic acid and 200 mg of Tween-80 and concentrated using N_2_. Then, 50 mL of oxygenated water was added and mixed. The emulsion obtained was freshly prepared before each experiment. A volume of 40 mL of VZ-EO (10 *μ*g/mL) or positive control (10 *μ*g/mL BHA) was added to 960 mL of the *β*-carotene/linoleic acid emulsion. An equal amount of methanol was used for the control. The reaction mixture was maintained at 50°C, and the absorbance was measured at 470 nm. The absorbances of all of the samples were read immediately (at the zero time point) and every 30 min up to 180 min.

### 2.3. Gas Chromatography and Mass Spectrometry Analysis

The essential oil was analyzed through gas chromatography-mass spectrometry (GC-MS) to identify its constituents. The GC-MS (GCMS-QP2010 Plus, Shimadzu, Japan) was equipped with a Forte ID-BPX5 column (30.0 m × 0.25 mm i.d., 0.25 *μ*m film thickness, SGE, AU), and the injector temperature was maintained at 250°C. Helium (He) was used as the carrier gas at a flow rate of 1 mL/min, and the split ratio was set to 1 : 100. The initial oven temperature was maintained at 50°C for 5 min and then programmed to increase at a rate of 5°C/min to 150°C and then at a rate of 10°C/min to 300°C. The mass spectrometry conditions were as follows: scan range, 40–350 amu; ion source temperature, 230°C; and interface temperature, 250°C. The essential oil constituents were identified by comparing the retention times and retention indices of the chromatographic peaks with a standard library from the National Institute of Standards and Technology (NIST) MS spectral database (version, 2005) and by comparing the measured Kovats index (KI) to a homologous series of* n*-alkanes (C_5_–C_26_).

### 2.4. Cell Cultures

The B16 murine melanoma cell line was purchased from the Bioresource Collection and Research Center (BCRC; Hsinchu, Taiwan). The B16 cells were cultured in DMEM supplemented with 10% FBS, 2 mM glutamine, 100 mg/mL streptomycin, and 100 U/mL penicillin. The cells were maintained in a humidified 5% CO_2_ incubator at 37°C and were subcultured every 3 to 4 days to maintain logarithmic growth.

### 2.5. Cell Viability

The B16 cells were seeded in a 96-well plate at a density of 5 × 10^3^ cells/well. After the plate was incubated for 24 h, different concentrations of the samples and 10 nM *α*-MSH were added to each well; the plate was then incubated for an additional 72 h. The cell viability was then determined using the improved MTT assay [13].

### 2.6. Melanin Content Assay

The method described by Thanigaimalai et al. [14] was used to determine the melanin content of the B16 cells. These cells were incubated with various concentrations of VZ-EO (2.5, 5.0, 10, or 20 *μ*g/mL) and were subsequently cotreated with 10 nM *α*-MSH for 72 h. After this treatment, the cells were dissolved in a 1 M NaOH 10% DMSO solution and incubated at 90°C to solubilize the melanin. The total melanin in each cell suspension was determined by the absorbance of each suspension at 405 nm. The melanin content was calculated by interpolating the results onto a standard curve that was generated by the absorbance of known concentrations of synthetic melanin and correcting the total amounts of protein that are present in the cell lysate supernatants.

### 2.7. Cellular Tyrosinase Activity Assay

The cellular tyrosinase activity was assayed based on the DOPA oxidase activity using the method described by Seo et al. [15]. The B16 cells were incubated with various concentrations of VZ-EO (2.5, 5.0, 10, or 20 *μ*g/mL) and were subsequently cotreated with 10 nM *α*-MSH for 72 h. At the end of this treatment, the cells were sonicated with phosphate buffer (pH 6.8) containing 1 mM PMSF. The lysates were clarified through centrifugation at 10,000 ×g for 10 min. After the protein in each lysate was quantified and protein concentration was adjusted with lysis buffer, 300 *μ*L of each lysate was mixed with 700 *μ*L of 5 mM L-DOPA. This mixture was incubated for 1 h at 37°C, and the absorbance was then measured spectrophotometrically at 475 nm.

### 2.8. Western Blot Assay

The B16 cells were incubated with various concentrations of VZ-EO (2.5, 5.0, 10, or 20 *μ*g/mL) and were subsequently cotreated with 10 nM *α*-MSH for 72 h. After this treatment, the cells were sonicated with phosphate buffer (pH 6.8) containing 1 mM PMSF, and the lysates were collected and then quantified. For western blotting, each well was loaded with 20 ng of protein, resolved by 12% SDS PAGE, and then electrotransferred onto a polyvinylidene difluoride (PVDF) membrane using a Bio-Rad Mini-Protean II apparatus (Bio-Rad Laboratories, Carlsbad, CA, USA). The blots were subsequently incubated with goat antityrosinase antibody or rabbit anti-*β*-actin antibody as primary antibodies, and the immune complexes were visualized using the ECL reagent. The bands were scanned and then quantified by measuring the optical densities using the VIpro Platinum 1.1 software (Version 12.9; UVItec, UK).

### 2.9. Measurement of Lipid Peroxide and Glutathione (GSH) Levels and Glutathione Peroxidase (GPX), Superoxide Dismutase (SOD), and Catalase (CAT) Activities

B16 cells were incubated with various concentrations of VZ-EO (2.5, 5.0, 10, or 20 *μ*g/mL) and subsequently cotreated with 10 nM *α*-MSH for 72 h. At the end of the treatment, the cells were harvested and sonicated with phosphate buffer (pH 6.8) containing 1 mM PMSF to obtain cell homogenates. The thiobarbituric acid-reactive substances (TBARS) method was used to estimate the cellular malondialdehyde (MDA) levels by measuring the absorbance at 535 nm with a spectrophotometer [16]. The cellular GSH was reduced with a dithiothreitol/phosphate solution and derivatized with MbBr prior to HPLC analysis [17].

With respect to the GPX activity, one unit of GPX activity was defined as the amount of enzyme that oxidized 1 nM NADPH per minute, as measured by the absorbance at 340 nm [18]. The superoxide dismutase (SOD) activity was determined spectrophotometrically at 325 nm by determining the SOD-mediated decrease in the rate of pyrogallol autoxidation under alkaline conditions [19]. One unit of SOD activity was defined as the amount of enzyme that inhibited the rate of pyrogallol oxidation. The catalase (CAT) activity was analyzed by measuring the decrease in the absorbance of H_2_O_2_ at 240 nm. One unit of CAT activity was defined as the amount of enzyme that decomposed 1.0 *μ*M H_2_O_2_ per minute [20]. The specific activities of SOD, CAT, and GPX are expressed in terms of units/mg protein.

### 2.10. Statistical Analysis

All of the measurements were conducted at least three times with three different sample preparations. All the data were expressed as the means ± standard deviation (SD). The analysis of variance was performed using the SPSS software (version 16.0; SPSS Inc., USA). One-way ANOVA and Scheffe's method were used to determine the differences between the means, and differences with *P* < 0.05 were considered to be statistically significant.

## 3. Results and Discussion

### 3.1. Antioxidant Activity of VZ-EO

**Figure 1:**
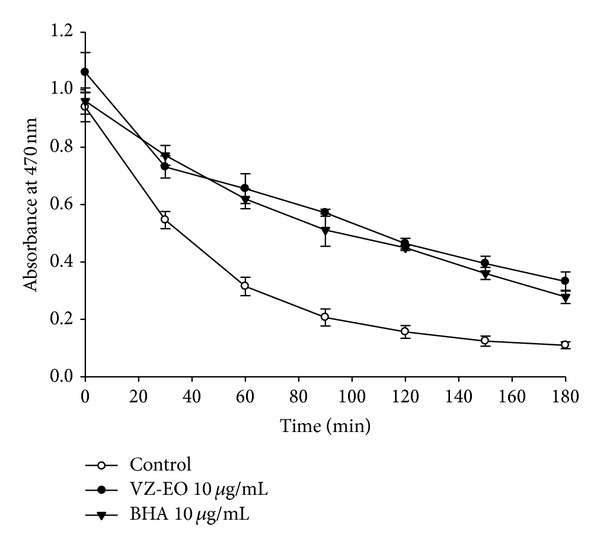
The antioxidant activity of VZ-EO assessed using the *β*-carotene/linoleic acid bleaching method. The activity was measured by the changes in the absorbance at 470 nm. The data are the means ± S.D. (*n* = 3).

The* in vitro* inhibitory activity of VZ-EO on lipid peroxidation was first established in this study through the *β*-carotene bleaching method. In the absence of an antioxidant, *β*-carotene undergoes rapid decolorization because it is attacked by free linoleic acid radicals. The results are shown in Figure 1. Compared with the control group, 10 *μ*g/mL VZ-EO can markedly slow down the decrease in the absorbance at 470 nm from 0 to 180 min. Moreover, its antioxidative performance on lipid peroxidation is comparable to that of the potent antioxidant BHA (Figure 1). Therefore, we confirmed that VZ-EO exhibits significant antioxidant activity on lipid peroxidation, and this result is similar to the findings reported by several earlier studies [10, 11].

### 3.2. Effect of VZ-EO on Cell Viability and Melanin Content in *α*-MSH-Stimulated B16 Cells

**Figure 2:**
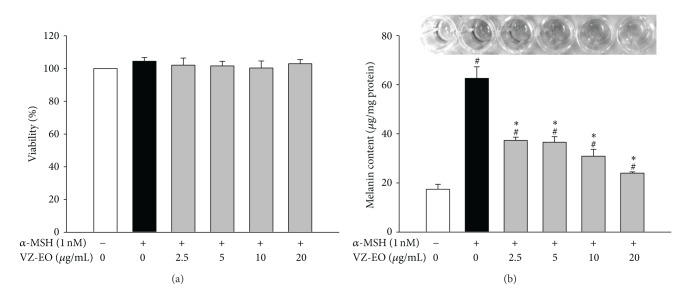
Effect of VZ-EO on the cell viability (a) and melanin content (b) in *α*-MSH-stimulated B16 cells. The data are means ± S.D. (*n* = 3). # indicates a significant difference (*P* < 0.05) compared with the control group; ∗ indicates a significant difference (*P* < 0.05) compared with the *α*-MSH-treated group.

To evaluate the effect of VZ-EO on *α*-MSH-stimulated B16 cells and to determine appropriate concentrations of VZ-EO for the subsequent analyses, the standard MTT assay was used to test the effect of VZ-EO on the B16 cell viability, and the results are shown in Figure 2(a). All of the tested concentrations (from 2.5 to 20 *μ*g/mL) of VZ-EO did not alter the proliferation of the *α*-MSH-stimulated B16 cells. Thus, all of the tested concentrations of VZ-EO were then used for the following experiments.

The effect of VZ-EO on the melanin content in *α*-MSH-stimulated B16 cells was tested and is shown in Figure 2(b). The results demonstrated that VZ-EO can suppress melanin production in *α*-MSH-stimulated B16 cells. The melanin content in the *α*-MSH-stimulated group increased to approximately 62 *μ*g/mg protein. As the concentration of VZ-EO increased from 2.5 to 20 *μ*g/mL, the melanin contents of the *α*-MSH-stimulated B16 cells treated with VZ-EO were reduced to only 38 to 24 *μ*g/mg protein (Figure 2(b)). Therefore, this is the first demonstration that VZ-EO can markedly decrease the *α*-MSH-induced melanin production in B16 cells.

### 3.3. Effect of VZ-EO on Cellular Tyrosinase Activity and Tyrosinase Protein Expression in *α*-MSH-Stimulated B16 Cells

**Figure 3:**
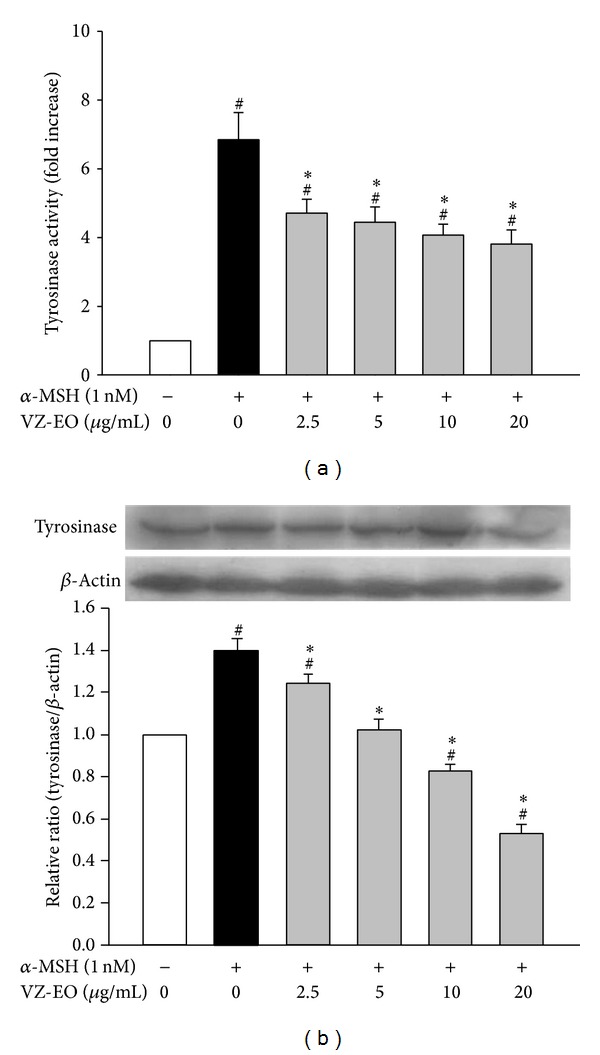
Effect of VZ-EO on cellular tyrosinase activity (a) and protein expression (b) in *α*-MSH-stimulated B16 cells. The data are the means ± S.D. (*n* = 3). # indicates a significant difference (*P* < 0.05) compared with the control group; ∗ indicates a significant difference (*P* < 0.05) compared with the *α*-MSH-treated group.

Both eumelanin and pheomelanin are derived from the same precursor amino acid, namely, L-tyrosine, through tyrosinase. Thus, tyrosinase is recognized as the rate-limiting enzyme in melanin biosynthesis in melanocytes [1]. To verify the effect of VZ-EO on melanin production, we tested the cellular tyrosinase activity in *α*-MSH-stimulated B16 cells, and the results are shown in Figure 3(a). Similar to the results obtained from the melanin content assay that are shown in Figure 2(b), the activity of tyrosinase in B16 cells was induced by *α*-MSH and reduced by treatment with VZ-EO. VZ-EO concentrations of 10 to 20 *μ*g/mL can strongly decrease the tyrosinase activities to approximately half of that obtained in the *α*-MSH-stimulated cells (Figure 3(a)).

To further verify the control point of VZ-EO on B16 melanin production, we analyzed the protein expression of tyrosinase by western blot analysis, and the results are displayed in Figure 3(b). The results clearly demonstrate that VZ-EO can decrease the *α*-MSH-induced increase in tyrosinase expression in B16 cells in a dose-dependent manner. In fact, treatment with 10 and 20 *μ*g/mL VZ-EO can decrease the expression of tyrosinase to 81 and 52% compared with the unstimulated control cells (Figure 3(b)). Therefore, we can hypothesize that the antimelanogenic activity of VZ-EO is implemented through the downregulation of both the activity and the protein level of tyrosinase and that this downregulation results in dose-dependent decreases in the melanin content of cells. Based on the aforementioned results, VZ-EO suppresses the *α*-MSH-stimulated melanogenesis in murine B16 melanoma cells through noncytotoxic mechanisms that involve the inhibition of tyrosinase activity and the downregulation of tyrosinase expression.

### 3.4. Effect of VZ-EO on MDA Production, GSH Levels, and Antioxidant Enzyme Activities in *α*-MSH-Stimulated B16 Cells

The oxidation of L-DOPA generates a highly reactive intermediate that is further oxidized to form melanin through a radical-coupling pathway. In fact, melanogenesis has been reported to involve not only the production of hydrogen peroxide (H_2_O_2_) by means of enzymatic and nonenzymatic reactions but also the subsequent generation of other reactive oxygen species (ROS), which cause oxidative stress for melanocytes [21]. Oxidative stress is considered the result of an imbalance between oxidants and antioxidants within the cell. Additionally, *α*-MSH-induced melanogenesis is associated with ROS generation [22]. Therefore, antioxidants, tyrosinase inhibitors, and ROS scavengers may downregulate melanogenesis. Many previous studies have reported that various antioxidants, including gallic acid [23], curcumin [3], and ascorbic acid [24], may exhibit antimelanogenic effects. Therefore, we investigated whether exposure to VZ-EO would reduce the oxidative stress in *α*-MSH-stimulated cells.

**Figure 4:**
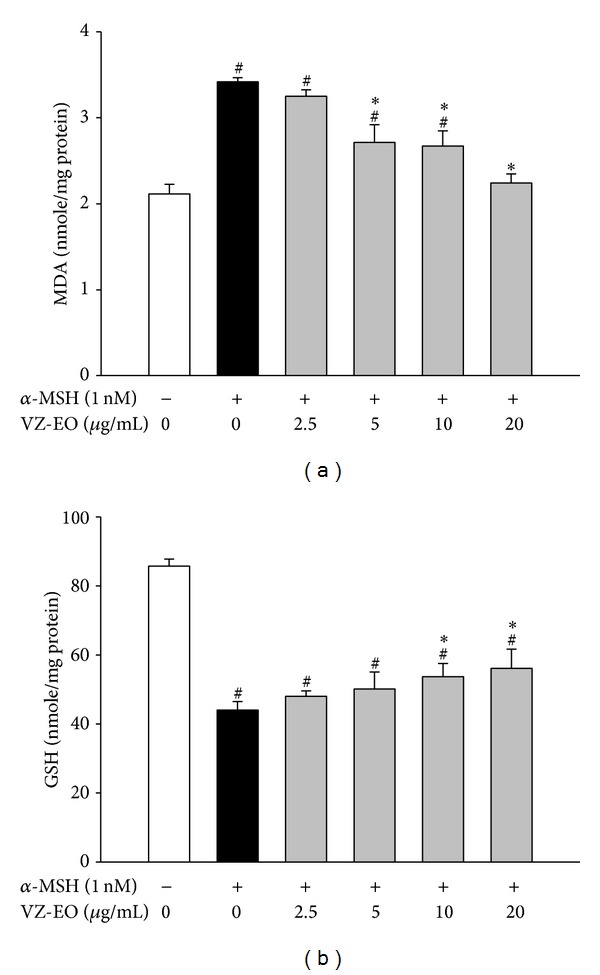
Effect of VZ-EO on the MDA (a) and GSH (b) levels in *α*-MSH-stimulated B16 cells. The data are the means ± S.D. (*n* = 3). # indicates a significant difference (*P* < 0.05) compared with the control group; ∗ indicates a significant difference (*P* < 0.05) compared with the *α*-MSH-treated group.

First, the level of lipid peroxidation was measured based on the production of MDA to determine the cellular oxidative stress induced by *α*-MSH. As shown in Figure 4(a), the MDA level in *α*-MSH-stimulated B16 cells was increased to 3.4 nmol/mg protein. However, VZ-EO (at concentrations from 2.5 to 20 *μ*g/mL) reduces the cellular MDA levels in a dose-dependent manner. Treatment with 20 *μ*g/mL VZ-EO decreased the *α*-MSH-stimulated MDA levels to a level that was almost the same as that observed in unstimulated cells. The level of GSH is important for maintaining the cellular redox status and plays an important role in the inhibition of melanogenesis [25]. Our data indicated that the GSH level in B16 cells was reduced by *α*-MSH treatment to only 43.7 nmol/mg protein. However, increasing concentrations of VZ-EO noticeably improved the cellular GSH levels (Figure 4(b)). Although the GSH levels in the VZ-EO-treated B16 cells did not return to the basal level, our results demonstrate that VZ-EO has the ability to decrease the *α*-MSH-activated oxidative stress in B16 cells.

**Figure 5:**
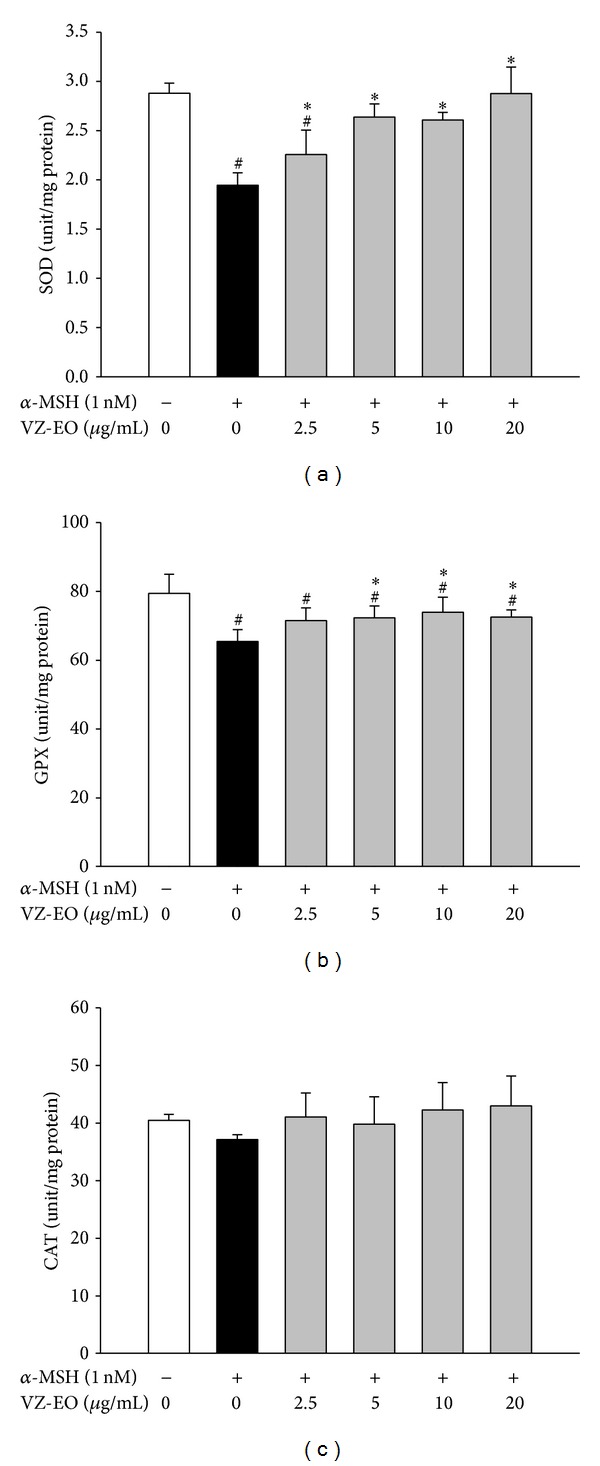
Effect of VZ-EO on the activities of SOD (a), GPX (b), and CAT (c) in *α*-MSH-stimulated B16 cells. The data are the means ± S.D. (*n* = 3). # indicates a significant difference (*P* < 0.05) compared with the control group; ∗ indicates a significant difference (*P* < 0.05) compared with the *α*-MSH-treated group.

Various antioxidant enzymes, such as GPX, SOD, and CAT, play important roles in maintaining the redox homeostasis within cells. A previous study revealed that gallic acid exhibits a protective effect on UV-mediated melanogenesis through improvement of the GSH-related antioxidant defenses [26]. Amikacin, which is an antibiotic derived from kanamycin, can modulate melanogenesis in melanocytes by the regulation of the antioxidant defense system, including the activities of SOD, GPX, and CAT [27]. To further evaluate the antioxidant activity of VZ-EO, we analyzed the activities of these important cellular antioxidative enzymes in *α*-MSH-stimulated B16 cells. The results are shown in Figure 5. The activities of all of the tested antioxidative enzymes in the *α*-MSH-treated cells were reduced. In addition, all of the tested antioxidative enzyme activities returned to their normal levels in *α*-MSH-stimulated B16 cells treated with 20 *μ*g/mL VZ-EO (Figure 5). Although the effects of both *α*-MSH and VZ-EO on the CAT activity in B16 cells were not obvious (Figure 5(c)), the results confirmed that the effect of VZ-EO on the activity of CAT was similar to the effect observed in the activities of SOD (Figure 5(a)) and GPX (Figure 5(b)). Therefore, we hypothesized that VZ-EO can increase the activities of cellular SOD, GPX, and CAT to suppress the oxidative stress induced by *α*-MSH in B16 cells.

**Figure 6:**
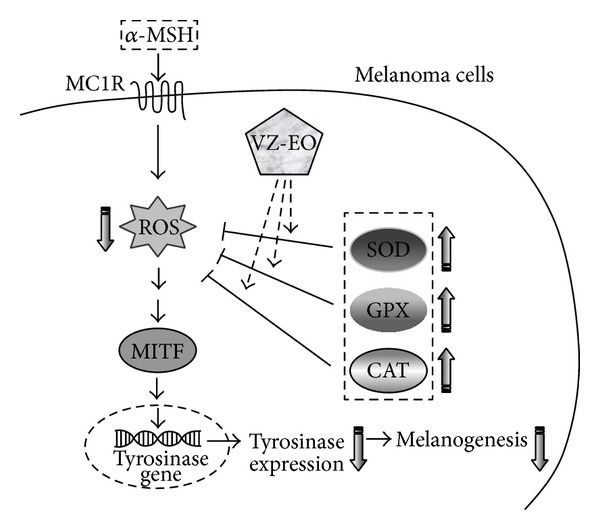
Proposed mechanisms for the effect of VZ-EO on melanogenesis in melanoma cells.

Therefore, our data demonstrate that depletion of the GSH content and aggravation of oxidative stress could be involved in the melanogenesis response induced by *α*-MSH in B16 melanoma cells. VZ-EO can repress the oxidative stress and lipid peroxidation in *α*-MSH-stimulated cells because this treatment increases both the levels of GSH and the activities of SOD and GPX within these cells and thereby reduces oxidant formation. Based on our results, we conclude that VZ-EO is able to suppress the *α*-MSH-induced melanogenesis through tyrosinase inactivation and the simultaneous suppression of oxidative stress in B16 melanoma cells. The proposed mechanism is shown in Figure 6.

### 3.5. Analysis of the Components of VZ-EO

The functions of an essential oil are often provided by its complex composition. Therefore, the activity of such functional ingredients cannot be easily explained by a few major compounds. However, to further clarify the effects of VZ-EO, the chemical composition of VZ-EO was also determined and is shown in Table 1. As shown in Table 1, the most abundant component of VZ-EO is cedr-8-en-13-ol (12.4%). Other major compounds of VZ-EO are *α*-amorphene (7.80%), *β*-vatirenene (5.94%), *α*-gurjunene (5.91%), and dehydroaromadendrene (5.45%). A previous study conducted by Tepe et al. [28] reported that the essential oil of* Peucedanum longifolium* contains 33.74% cedr-8-en-13-ol and exhibited a strong ability to inhibit lipid peroxidation. Moreover, the antioxidant and biological functions of* V. zizanioides* have been described by some previous studies [10, 11]. Therefore, the results obtained in this study might explain insight into the antioxidant function and antimelanogenic activity of VZ-EO.

## 4. Conclusions

In summary, VZ-EO exhibits powerful antioxidative activity on lipid peroxidation to moderate the bleaching of *β*-carotene and to maintain the cellular GSH level. VZ-EO can markedly decrease melanin production in *α*-MSH-stimulated B16 cells. The effect of VZ-EO on the melanogenesis induced by *α*-MSH in B16 cells is achieved through the suppression of cellular tyrosinase expression. The function of VZ-EO on melanogenesis might result from its potent antioxidative ability, which is reflected by the restoration of the cellular SOD, GPX, and CAT activities in *α*-MSH-stimulated B16 cells. The most abundant component of VZ-EO is cedr-8-en-13-ol (12.4%), which has the ability to inhibit lipid peroxidation strongly. Therefore, VZ-EO has the potential to become an ingredient in future hypopigmentation drugs, foods, and cosmetics.

## Figures and Tables

**Table 1:** GC-MS analysis of the essential oil from *Vetiveria zizanioides* (VZ-EO).

Compound	Rt	KI	Area (%)	M.f.
2,3,5,5,8,8-Hexamethyl-cycloocta-1,3,6-triene	23.19	1314	2.28	C_14_H_22_
1,5,9,9-Tetramethyl-2-methylene-spiro[3.5]non-5-ene	24.08	1328	3.96	C_14_H_22_
(+)-Sativen	25.51	1339	2.83	C_15_H_24_
4,8,8-Trimethyl-2-methylene-4-vinylbicyclo[5.2.0]nonane	25.62	1407	4.53	C_15_H_24_
*α*-Amorphene	26.05	1440	7.80	C_15_H_24_
2-Isopropenyl-1,3,5-trimethylbenzene	26.14	1465	2.43	C_12_H_16_
*α*-Gurjunene	26.22	1479	5.91	C_15_H_24_
*β*-Vatirenene	26.30	1489	5.94	C_15_H_22_
*δ*-Cadinene	26.55	1499	2.57	C_15_H_24_
*β*-Guaiene	26.93	1523	4.28	C_15_H_24_
Dehydroaromadendrene	27.05	1545	5.45	C_15_H_22_
Cubenol	28.82	1580	2.09	C_15_H_26_O
(+)-Ledene	29.28	1605	4.77	C_15_H_24_
Epiglobulol	29.42	1632	2.21	C_15_H_26_O
Widdrol	29.90	1651	2.13	C_15_H_26_O
6-Isopropenyl-4,8a-dimethyl-1,2,3,5,6,7,8,8a-octahydro-naphthalen-2-ol	30.04	1690	1.97	C_15_H_24_O
3-(2-Isopropyl-5-methylphenyl)-2-methylpropionic acid	30.17	1745	3.17	C_14_H_20_O_2_
Cedr-8-en-13-ol	30.67	1769	12.36	C_15_H_24_O
Ethyl 4-(4-methylphenyl)-4-pentenoate	31.06	1804	2.12	C_16_H_20_O_3_
Isovellerdiol	31.11	1842	2.38	C_15_H_24_O_2_
*α*-Curcumene	31.16	1867	2.44	C_15_H_22_
3,3,8,8-Tetramethyl-tricyclo[5.1.0.0(2,4)]oct-5-ene-5-propanoic acid	31.38	1890	4.82	C_15_H_22_O_2_
Solavetivone	31.46	1906	4.20	C_15_H_22_O
3,8-Dimethyl-4-(1-methylethylidene)-2,4,6,7,8,8a-hexahydro-5(1H)-azulenone	31.82	1925	4.89	C_15_H_22_O
(−)-Spathulenol	31.88	1938	2.47	C_15_H_24_O

Rt: retention time (min); KI: Kovats index; M.f.: molecular formula.
